# Experimental Acute Exposure to Thirdhand Smoke and Changes in the Human Nasal Epithelial Transcriptome: A Randomized Clinical Trial

**DOI:** 10.1001/jamanetworkopen.2019.6362

**Published:** 2019-06-28

**Authors:** Giovanna L. Pozuelos, Meenakshi S. Kagda, Suzaynn Schick, Thomas Girke, David C. Volz, Prue Talbot

**Affiliations:** 1 Department of Molecular, Cell and Systems Biology, University of California, Riverside; 2 Department of Medicine, University of California, San Francisco; 3 Department of Botany and Plant Sciences, University of California, Riverside; 4 Department of Environmental Sciences, University of California, Riverside

## Abstract

**Importance:**

No previous studies have shown that acute inhalation of thirdhand smoke (THS) activates stress and survival pathways in the human nasal epithelium.

**Objective:**

To evaluate gene expression in the nasal epithelium of nonsmoking women following acute inhalation of clean air and THS.

**Design, Setting, and Participants:**

Nasal epithelium samples were obtained from participants in a randomized clinical trial (2011-2015) on the health effects of inhaled THS. In a crossover design, participants were exposed, head only, to THS and to conditioned, filtered air in a laboratory setting. The order of exposures was randomized and exposures were separated by at least 21 days. Ribonucleic acid was obtained from a subset of 4 healthy, nonsmoking women.

**Exposures:**

By chance, women in the subset were randomized to receive clean air exposure first and THS exposure second. Exposures lasted 3 hours.

**Main Outcomes and Measures:**

Differentially expressed genes were identified using RNA sequencing with a false-discovery rate less than 0.1.

**Results:**

Participants were 4 healthy, nonsmoking women aged 27 to 49 years (mean [SD] age, 42 [10.2] years) with no chronic diseases. A total of 389 differentially expressed genes were identified in nasal epithelium exposed to THS, while only 2 genes, which were not studied further, were affected by clean air. Enriched gene ontology terms associated with stress-induced mitochondrial hyperfusion were identified, such as respiratory electron transport chain (*q* = 2.84 × 10^−3^) and mitochondrial inner membrane (*q* = 7.21 × 10^−6^). Reactome pathway analysis identified terms associated with upregulation of DNA repair mechanisms, such as nucleotide excision repair (*q* = 1.05 × 10^−2^). Enrichment analyses using ingenuity pathway analysis identified canonical pathways related to stress-induced mitochondrial hyperfusion (eg, increased oxidative phosphorylation) (*P* = .001), oxidative stress (eg, glutathione depletion phase II reactions) (*P* = .04), and cell survival (*z* score = 5.026).

**Conclusions and Relevance:**

This study found that acute inhalation of THS caused cell stress that led to the activation of survival pathways. Some responses were consistent with stress-induced mitochondrial hyperfusion and similar to those demonstrated previously in vitro. These data may be valuable to physicians treating patients exposed to THS and may aid in formulating regulations for the remediation of THS-contaminated environments.

## Key Points

**Question:**

Does acute inhalation of thirdhand smoke alter the transcriptome of the human nasal epithelium?

**Findings:**

This randomized clinical trial exposed 4 healthy, nonsmoking women to clean air, which altered the expression of only 2 genes. When the same women were exposed to thirdhand smoke at least 21 days later, 389 genes associated with cell stress and survival pathways were differentially expressed, and many affected genes were associated with increased mitochondrial activity, oxidative stress, DNA repair, cell survival, and inhibition of cell death.

**Meaning:**

These results suggest that acute exposure to thirdhand smoke stresses the human nasal epithelium, a finding that may be valuable to physicians treating exposed patients.

## Teaser

This randomized clinical trial compares changes in the transcriptome of the human nasal epithelium in healthy, nonsmoking women who were first exposed to clean air and later exposed to thirdhand smoke.

## Introduction

Thirdhand smoke (THS) is a subset of chemicals in secondhand cigarette smoke (sidestream smoke emitted by a burning cigarette and exhaled mainstream smoke) that sticks to indoor surfaces and persists after active smoking has occurred.^1,2^ Chemicals in THS accumulate and can react with other compounds or can be reemitted into the environment.^1,2,3^ Nonsmokers can be exposed to chemicals in THS months or even years after smoking has stopped.^3^ Many THS chemicals are toxic volatile and semivolatile organic compounds.^2,3,4^ Nicotine, a major chemical in THS, has a high affinity for surfaces^3^ and can react with ambient nitrous acid to form tobacco-specific nitrosamines, some of which are carcinogens.^5,6^ Nicotine-derived nitrosamines in THS include 4-(methylnitrosamino)-1-(3-pyridinyl)-1-butanone, and N-nitrosonornicotine,^5,6^ which are also found in secondhand smoke and have been associated with the development of lung cancer.^7^ Ozone can also react with nicotine to form formaldehyde, a known human carcinogen.^8^

Owing to the presence of these and other hazardous chemicals in THS, such as acrolein, it is important to understand whether there is a correlation between exposure to THS and human health, especially in nonsmokers. Previous studies^9,10^ have demonstrated that exposure of human cell lines to THS extracts for 24 hours increased DNA strand breaks and oxidative DNA damage. Mouse neural stem cells undergo blebbing, fragmentation, cytoskeletal disruption, and vacuolization when treated with extracts of THS.^11^ Thirdhand smoke is also associated with stress-induced mitochondrial hyperfusion (SIMH), which is accompanied by increased mitochondrial membrane potential, adenosine triphosphate (ATP) production, and reactive oxygen species (ROS).^12^ During SIMH, punctate mitochondria fuse and form tubular networks that allow exchange of molecules, including mitochondrial DNA, as a survival mechanism.^13^ Acrolein has been identified as a THS chemical that inhibits cell proliferation.^11^ In a metabolomics study using male germ cells, THS exposure was associated with downregulation of several molecular pathways, including nucleic acid metabolism and ammonia metabolism, and upregulation of glutathione metabolism.^14^

Thirdhand smoke is also associated with adverse health effects in mice. Three-week old mice that were housed for 6 months in cages containing a THS-impregnated fabric and bedding showed an increase in inflammatory cytokines in lung tissue, impaired wound healing, and were hyperactive compared with controls.^15^ Adult mice developed insulin resistance as a consequence of oxidative stress associated with THS and showed increased blood glucose levels, increased serum insulin levels, and accumulation of fat in viscera.^16^ Oxidative stress in skeletal muscle and accumulation of hydrogen peroxide accompanied by low catalase activity was observed in long-term exposed mice.^17^ After THS exposure, neonatal mice had significantly more eosinophils, increased platelet volume, lower hematocrit levels, and decreased mean cell volume than controls, while adult exposed mice had a significant increase in the percentage of B-cells and a decrease in myeloid cells.^18^

Elimination of THS can be challenging, as it persists in houses previously owned by smokers even after 2 months of vacancy.^19^ Cars previously owned by smokers also retain THS, and new owners may be at risk of exposure.^20^ Common household fabrics retained THS chemicals 19 months after smoking had occurred.^4^ Individuals absorb nicotine through their skin while wearing THS-exposed clothes.^21^ Moreover, infants whose mothers smoked outdoors had much higher levels of urine cotinine, a nicotine metabolite, than infants of nonsmoking parents.^22^ Other examples of the persistence of THS have been reviewed recently.^2^

Although these prior studies demonstrate that humans are at risk of exposure to THS, the molecular effects of such exposure on humans have not been investigated. The purpose of this study was to evaluate the effects of inhalation of THS chemicals on gene expression in humans. Nasal epithelial cells were collected from nonsmokers before and after 3 hours of exposure to either clean air or to THS, subjected to RNA sequencing, and analyzed for differential expression of genes (DEG). Significant changes in gene expression were found following THS exposure, but not exposure to clean air.

## Methods

### Ethics

The study was approved by University of California, San Francisco institutional review board. Written informed consent was given by all participants. This study followed the Consolidated Standards of Reporting Trials (CONSORT) reporting guideline. A trial protocol including details of participant recruitment, written informed consent, screening, selection, compensation, and involvement in the study is available in Supplement 1. The RNA sequencing analysis was approved by the University of California at Riverside institutional review board.

### Study Population, Generation of THS, and THS Exposure

**Figure 1.**
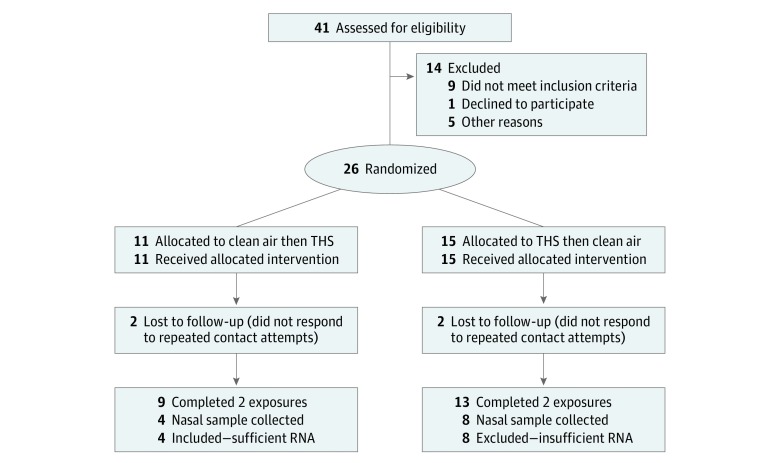
CONSORT Flow Diagram of Parent Study and Subset Sample Of 26 participants included in the parent study, nasal epithelial samples from 4 had sufficient RNA to be included in the subset sample.

The protocol for the primary study during which the nasal epithelial cell samples were collected appears in the CONSORT flow diagram (Figure 1) and Supplement 1. It was conducted at the University of California, San Francisco, between 2011 and 2015. Briefly, 26 healthy nonsmokers who were not exposed to secondhand cigarette smoke in daily life were exposed, head-only, to THS aerosol and to conditioned, filtered air for 3 hours using an exposure chamber described previously.^23^ Of these 26 individuals, 13 (8 women and 5 men) had nasal epithelial cell samples collected before and after each exposure. Nasal epithelial samples were collected from the anterior, inferior turbinate using small, sterile plastic curettes (RhinoPro; Arlington Scientific, Inc). These samples were immediately placed in RNAlater (Qiagen) and shipped frozen to the University of California, Riverside, where RNA extraction and subsequent analyses were performed.

### RNA Isolation

Ribonucleic acid was isolated from human nasal samples using RNeasy Micro Kits (Qiagen) and stored at −80 °C. We quantified RNA using a NanoDrop ND-1000 Spectrophotometer (Thermo Fisher Scientific). Samples from 4 participants had RNA concentrations greater than 3 ng/μL, and these were used for subsequent analysis. Frozen RNA samples were shipped to Cofactor Genomics for library preparation and sequencing.

### RNA Sequencing

Cofactor Genomics performed quality control on RNA samples, and RNA integrity was determined using a the Agilent 2100 Bioanalyzer. Samples with RNA integrity numbers between 8 and 10 were used for library construction. Total RNA was reverse-transcribed using an Oligo (dT) primer (Co-Factor), and limited cDNA amplification was performed using the SMARTer Ultra Low Input RNA Kit for Sequencing–v4 (Takara Bio USA, Inc). Full-length cDNA was fragmented and tagged, followed by limited polymerase chain reaction enrichment to generate the final cDNA sequencing library (Nextera XT DNA Library Prep; Illumina). Libraries were sequenced as single-end 75–base pair reads using an Illumina NextSeq500 following the manufacturer's instructions. Because the amount of nasal epithelium in each sample was very limited, we were not able to perform confirmatory quantitative polymerase chain reaction.

### Bioinformatics Analysis

FASTQ files obtained from Cofactor were processed on a high-performance computing cluster at the University of California, Riverside. The RNA sequencing analysis workflow implemented by systemPipeR^24^ was used to perform all the downstream data processing. Briefly, adapter sequences and low-quality tails were removed from the raw reads using the Trimmomatic package.^25^ The preprocessed reads were then aligned against the hg19 human reference genome in the University of California, Santa Cruz, Genome Browser with Tophat2 (version 2.0.14).^26,27^ Read counting was performed with the summarizeOverlaps function of the GenomicsAlignment package. Only unique reads overlapping the exonic gene regions were counted.^28^ Using a cutoff value of at least 1 read per kilobase per million of mapped reads averaged across all samples, raw expression counts of the remaining 10 938 genes passing this filter were used for differential expression analysis with EdgeR.^29^ Within each experimental group (group 1, 2, 3, and 4), the read counts from the 4 biological replicates were combined. For differential expression analysis, groups 1 and 2 (before and after clean air) and groups 3 and 4 (before and after THS) were treated as 2 separate experimental comparisons. Genes were considered to be DEGs if they had a false discovery rate (FDR) less than 0.1 by EdgeR. ClusterProfiler^30^ and ReactomePA^31^ packages were used to identify overrepresented GO terms and enriched Reactome pathways, respectively, as described in the package manual. Additionally, enrichment analyses of pathways were performed using the Ingenuity Pathway Analysis (IPA) software (Qiagen). Briefly, statistically significant transcripts were uploaded to IPA, and human homologs were automatically identified using the National Center for Biotechnology Information’s HomoloGene.

### Statistical Analysis

The EdgeR package was used to obtain log-fold changes, *P* values, and FDR scores (based on the Benjamini-Hochberg method). A gene was considered significantly differentially expressed if the FDR was less than 0.1. ClusterProfiler version 3.12.0 and ReactomePA version 1.28.0 packages used a Benjamini-Hochberg adjusted *P* value of less than .05 to identify significantly enriched Gene Ontology (GO) terms and Reactome pathways, respectively. Ingenuity Pathway Analysis used the Fisher 1-tailed exact test with a *P* value threshold of .05 to identify statistically significant pathways; the algorithm considered both direct and indirect relationships using the Ingenuity Knowledge Base (genes only) as the reference set. Analysis was performed using the EdgeR package in R statistical analysis software version 3.263 (R Project for Statistical Computing).

## Results

### Exposure to THS Altered Gene Expression in Human Nasal Epithelium

Participants were 4 healthy, nonsmoking women aged 27 to 49 years (mean [SD] age, 42 [10.2] years) with no chronic diseases. The samples collected were small and sufficient quantities of RNA for sequencing analysis could only be extracted from 4 women. By chance, these 4 participants had all been randomized to receive the clean air exposure first and THS exposure second; thus, we were unable to determine the effect of order on RNA expression. After processing RNA sequencing reads, data were analyzed to determine whether there were differences in gene expression in the groups exposed to either clean air (group 1 vs group 2) or THS (group 3 vs group 4) (eFigure 1 in Supplement 2). The data set consisted of approximately 10 000 genes, of which 2 and 389 were significantly differentially affected (FDR <0.10) in participants exposed to clean air and to THS, respectively (eTable 1 in Supplement 2). The 2 downregulated genes (hemoglobin, alpha 1 and hemoglobin, alpha 2) identified when participants were exposed to clean air had an absolute fold change of 8.2 and 8.7, respectively (eTable 2 in Supplement 2). No genes were significantly upregulated in the group exposed to clean air (eFigure 2 in Supplement 2). Because these results showed that wearing the respirator for 3.5 hours and inhaling clean air did not significantly impact gene expression, clean air was not studied further.

Nasal samples collected after THS exposure had a significant number of DEGs compared with samples collected before exposure (eTable 1 and eFigure 2 in Supplement 2). A total of 382 genes were significantly upregulated (FDR <0.1), while 7 were downregulated (eTable 1 in Supplement 2). The log_2_-fold changes for upregulated genes ranged from 2 to 7, while downregulated genes ranged from −2 to −9 (eTable 3 in Supplement 2). These data demonstrate that inhalation of THS for a relatively short time significantly altered gene expression in the human nasal epithelium.

### GO Term Enrichment Analysis

**Figure 2.**
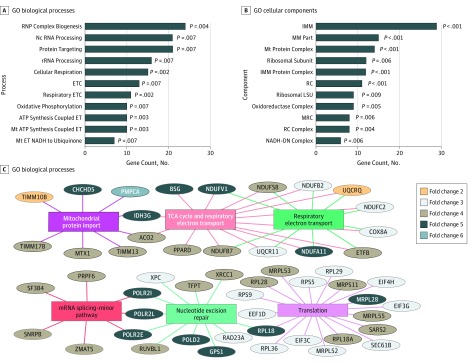
Gene Ontology (GO) and Reactome Pathway Enrichment Analysis of the Differentially Expressed Genes in Human Nasal Epithelium Exposed to Thirdhand Smoke Bar charts show the most highly enriched biological process (A) and cellular component (B) GO terms. Each bar represents the number of genes identified in our study that are associated with each process or component. All biological processes and cellular components identified had an adjusted *P* value for multiple testing less than .05. C, Network plot shows the top 6 enriched pathways and the associated genes using Reactome pathway analysis. Also shown are the approximate fold change values of each gene. The colored lines show the link between the genes and pathways identified. ATP indicates adenosine triphosphate; ET, electron transport; ETC, electron transport chain; IMM, mitochondrial inner membrane; LSU, large subunit; MM, mitochondrial membrane; MRC, mitochondrial respiratory chain; mRNA, messenger RNA; Mt, mitochondria; NADH-DN, nicotinamide adenine dinucleotide dehydrogenase complex; Nc, noncoding RNA; RC, respiratory chain; RNP, ribonucleoprotein; rRNA, ribosomal RNA; and TCA, trichloroacetic acid cycle.

We performed GO enrichment analysis on the upregulated DEGs to identify biological functions associated with THS (Figure 2A and B; eTable 4, eTable 5, and eTable 6 in Supplement 2). The GO database categorizes genes into different ontologies that represent biological knowledge.^32^ Our analysis identified 11 functions enriched within the biological processes, 13 within cellular components, and 1 within molecular function. All the processes were significantly enriched (*q* < 0.05) (eTable 4, eTable 5, and eTable 6 in Supplement 2). Most of the affected biological processes and cellular components in participants exposed to THS involved mitochondrial function or RNA metabolism. The top GO biological process terms included ribonucleoprotein complex biogenesis (GO:0022613), cellular respiration (GO:0045333), respiration electron transport chain (GO:0022904) (*q* = 2.84 × 10^−3^), and mitochondrial ATP synthesis coupled electron transport (GO:0042775) (Figure 2A). Most of the remaining GO biological processes included oxidative phosphorylation-related functions (eTable 4 in Supplement 2). The top enriched GO cellular components terms included mitochondria protein complex (GO:0098798), mitochondrial membrane part (GO:0044455), ribosomal subunit (GO:0044391), mitochondrial inner membrane (GO:0005743) (*q* = 7.21 × 10^−6^), respiratory chain (GO:0070469), large ribosomal subunit (GO:0015934), and respiratory chain complex (GO:0098803) (Figure 2B). All the remaining GO terms involved mitochondrial functions except for the 2 that were related to ribosomal subunit (eTable 5 in Supplement 2). No enriched GO terms could be identified for the downregulated genes in the THS experimental group, most likely owing to the small number of genes in this set.

### Reactome Enrichment Analysis

The Reactome enrichment analysis was used to further evaluate the upregulated DEGs after THS exposure. This analysis yielded a total of 25 pathways that were significantly enriched (eTable 7 in Supplement 2). The top 6 pathways (Figure 2C) included the citric acid cycle (R-HSA-1428517), respiratory electron transport (R-HSA-611105), translation (R-HSA-72766), mitochondrial protein import (R-HSA-1268020), mRNA splicing-minor pathway (R-HSA-72165), and nucleotide excision repair (R-HAS-5696398) (*q* = 1.05 × 10^−2^). Figure 2C shows the genes associated with each pathway and the overlap for those belonging to multiple pathways. Also shown are the approximate fold change values of each gene.

### Ingenuity Pathway Analysis

Ingenuity Pathway Analysis was also performed using upregulated genes in the THS-exposed group. The top pathways identified included sirtuin signaling pathways, EIF2 signaling, mitochondrial dysfunction, and oxidative phosphorylation (*P* = .001) (Table 1). Some pathways identified in IPA overlapped with those identified using Reactome enrichment analysis, including mitochondrial related pathways and DNA repair-related pathways. The top toxicological pathways identified included mainly processes related to mitochondrial activity, such as mitochondrial dysfunction, increases transmembrane potential of mitochondria and mitochondrial membrane, and decreases permeability transition of mitochondria and mitochondrial membrane. In addition, genes were linked to glutathione depletion phase II reactions (*P* = .04) (Table 1).

**Table 1.** Ingenuity Pathway Analysis–Enriched Pathways After Exposure to Thirdhand Smoke

Pathway	*P* Value	*P* Value		Genes, No.
		>.01	<.01	
Canonical pathways				
Sirtuin signaling pathway	.01	X		11
EIF2 signaling	.006		X	10
Mitochondrial dysfunction	.002		X	9
Oxidative phosphorylation	.001		X	8
Hereditary breast cancer signaling	.03	X		6
Oncostatin M signaling	.003		X	4
Nucleotide excision repair pathway	.003		X	4
Colanic acid building blocks biosynthesis	.002		X	3
Methionine degradation I (to homocysteine)	.005		X	3
Cysteine biosynthesis III (mammalia)	.006		X	3
Glutathione-mediated detoxification	.008		X	3
Superpathway of methionine degradation	.02	X		3
Serine biosynthesis	.003		X	2
Superpathway of serine and glycine biosynthesis I	.006		X	2
γ-glutamyl cycle	.02	X		2
UDP-*N*-acetyl-d-galactosamine biosynthesis I	.02	X		1
Spliceosomal cycle	.03	X		1
l-DOPA degradation	.03	X		1
GDP-l-fucose biosynthesis I (from GDP-D-mannose)	.03	X		1
Top toxicological pathways				
Mitochondrial dysfunction	.003		X	9
Increased transmembrane potential of mitochondria and mitochondrial membrane	.06	X		3
Decreased permeability transition of mitochondria and mitochondrial membrane	.006		X	2
Glutathione depletion phase II reactions	.04	X		2

Diseases and functions associated with the DEGs after THS exposure were identified by IPA (Table 2). These data were filtered and only functions with activated *z* scores that predict transcriptional activation or inhibition based on literature reports are presented (Table 2). The identified functions included decreased cell death and increased cell viability, homologous recombination, and cell proliferation. eFigure 3 in Supplement 2 shows upregulated genes associated with inhibition of cell death. The figure includes gene names and whether their expression could activate (orange lines) or inhibit (blue lines) cell death. For cell death, the majority of the upregulated genes predict inhibition (blue lines). Based on each gene’s biological role, IPA predicted that cell death had an activation *z* score of −3.117 (overall process decreased) (Table 2). Complementary to cell death, cell viability (*z* score = 5.026) (eFigure 4 in Supplement 2) and homologous recombination (*z* score = 2.828) (eFigure 5 in Supplement 2) both had increased activation states (Table 2).

**Table 2.** Disease and Function Annotations From Ingenuity Pathway Analysis

Categories	Diseases or Functions Annotation	Predicted Activation State	Activation *z* Score	Molecules, No.	*P* Value	*P* Value	
						>.01	<.01
Cell death and survival	Cell death	Decreased	−3.117	77	.002		X
Cell death and survival	Apoptosis	Decreased	−3.686	63	.001		X
Cell death and survival	Necrosis	Decreased	−2.641	59	.04	X	
Cell death and survival	Cell death of tumor cell lines	Decreased	−3.029	50	.03	X	
Cell death and survival	Apoptosis of tumor cell lines	Decreased	−2.617	41	.02	X	
Cell death and survival	Cell viability	Increased	5.026	38	.01	X	
Cell death and survival	Cell viability of tumor cell lines	Increased	4.59	32	.02	X	
Cell death and survival	Cell viability of breast cancer cell lines	Increased	3.094	10	.02	X	
Cell death and survival	Cell viability of blood cells	Increased	2.195	6	.02	X	
Cell death and survival	Cell viability of leukocytes	Increased	2.2	5	.03	X	
Infectious diseases	Viral Infection	Increased	5.315	54	.002		X
Infectious diseases	Infection by RNA virus	Increased	4.494	31	.03	X	
Infectious diseases	Infection of cells	Increased	4.594	29	.009		X
Infectious diseases	Infection by HIV-1	Increased	4.301	23	.02	X	
Infectious diseases	Replication of RNA virus	Increased	3.087	19	.004		X
Infectious diseases	Infection of cervical cancer cell lines	Increased	3.772	18	.01	X	
Infectious diseases	Replication of influenza A virus	Increased	2.824	13	.005		X
Cell cycle, DNA replication, recombination, and repair	Homologous recombination of cells	Increased	2.828	8	<.001		X
Cellular development, cellular growth and proliferation	Cell proliferation of breast cancer cell lines	Increased	2.811	18	.03	X	

## Discussion

The adverse health effects of THS have been studied in cultured cells and animal models,^2^ but to our knowledge similar investigations have not been previously performed in humans. Our study provides the first insight, to our knowledge, into the transcriptional responses of human respiratory epithelium to acute THS exposure. Remarkably, we found changes in gene expression in healthy nonsmokers following a 3-hour exposure to THS. The absence of an effect following clean air exposure provides evidence that the changes in gene expression following THS exposure are caused by THS per se and not by the respirator worn during exposure. Because gene expression in the nasal epithelium is similar to the bronchial epithelium,^33^ our data are also relevant to the cells deeper in the respiratory system.

Our analyses demonstrated that brief exposure to THS affected mitochondrial activity. We previously reported that cultured mouse neural stem cells undergo SIMH following exposure to THS extracts.^12^ This process was originally described during treatment of mouse embryonic fibroblasts with UV light and cell cycle inhibitors, such as actinomycin D.^13^ Stress-induced mitochondrial hyperfusion is characterized by fusion of mitochondria and subsequent increased production of ATP and superoxide.^12^ We found an enrichment in pathways and biological processes related to increased mitochondrial activity and oxidative stress after THS exposure, such as mitochondrial ATP synthesis coupled electron transport chain (GO:0042773), respiratory electron transport (R-HSA-611105), and oxidative phosphorylation (Table 1). Increased expression of these pathways is also consistent with an increase in ATP synthesis, as occurs in SIMH.^12^ Some genes related to the citric acid cycle were also upregulated, which could also increase ATP production. Several studies have shown that cigarette smoking also induces activation of mitochondrial pathways similar to those found in our study.^34,35,36^

While SIMH results in increased ATP production, it also increases ROS.^12,13^ Our IPA analysis showed that glutathione depletion phase II reactions were upregulated after THS exposure. Specifically, there was an increase in glutathione synthetase expression, which was also increased in a male germ cell line exposed to THS.^14^ This gene is part of the glutathione synthetase pathway, which scavenges ROS,^37^ suggesting the increase of the glutathione synthetase gene is a cellular response to high levels of ROS.

In prior studies, increased ROS was associated with oxidative stress and damage of proteins, lipids, and DNA,^38^ while THS treatment was correlated with DNA damage in vitro.^9,10^ Our IPA-based enriched pathway analysis included upregulation of the nucleotide excision repair pathway in participants exposed to THS. Two of the genes affected in this pathway included xeroderma pigmentosum group C and RNA polymerase II. The former is essential for recognition of DNA damage and plays a role in the early steps of the nucleotide excision repair pathway.^39^ Upregulation of RNA polymerase II has also been associated with a response to increased DNA damage.^40^ Ingenuity Pathway Analysis also identified an increased activation of homologous recombination. This pathway provides a repair mechanism for double-stranded DNA breaks.^41^ Activation of the DNA repair pathways is also a cellular mechanism to facilitate survival.^42^ In addition, an in vitro study showed that THS induces oxidation of mitochondrial proteins.^12^ The increase in ROS as evidenced by upregulation of ROS scavenging genes in our data could also result in oxidation of mitochondrial proteins by high local concentrations of superoxide.

Our data further demonstrate an overall increase in processes related to cell viability, which includes some genes involved in cell proliferation. Our results are consistent with previous in vitro studies showing increased proliferation of cultured mouse neural stem cells and human lung cancer cells exposed to THS extract.^12,43^ Nicotine, a major component of THS^4^ and a chemical in our exposure chamber, can activate alpha nicotinic acetylcholine receptors in normal human airway epithelial cells, leading to phosphorylation (activation) of serine/threonine kinase Akt, which is involved in many cellular survival pathways.^44^ Akt can be activated within minutes of exposure to nicotine or 4-(methylnitrosamino)-1-(3-pyridinyl)-1-butanone,^44^ further demonstrating that chemicals in THS could produce a rapid response. Nicotine is also associated with increased proliferation of human cancer cell lines by activating the α7 nicotinic acetylcholine receptors.^45^ Considering that nicotine stimulates cell proliferation,^45^ it is possible that nicotine in THS contributes to the increase in cell viability pathways that we observed.

Nicotine is also involved in inhibiting apoptosis.^46^ In our study, the increased expression of genes involved in inhibiting cell death (eFigure 3 in Supplement 2) may have been associated with nicotine, which was present in the THS at a concentration of 0.03 mg/L (to convert to micromoles per liter, multiply by 6.164). Consistent with our study, cells exposed to THS in vitro showed decreased expression in proapoptotic genes.^12^ The mechanism by which nicotine inhibits apoptosis has been studied in mouse liver cells.^47^ Activation of α7 nicotinic acetylcholine receptors in the mitochondrial outer membrane by nicotine inhibited hydrogen peroxide–induced apoptosis by impairing calcium ion accumulation in mitochondria and cytochrome C release.^47^ However, this suppression of cell death may be transitory. Bahl et al^12^ showed that cells exposed to THS for 30 days had a decrease in cell proliferation and lost mitochondrial membrane potential, indicating that cells were entering apoptosis.

### Limitations

This study has limitations. This is an initial study based on 4 participants. Future work should be done to determine whether similar data are obtained with a larger number of participants that includes both sexes. In addition, longer exposures to THS could be studied.

## Conclusions

**Figure 3.**
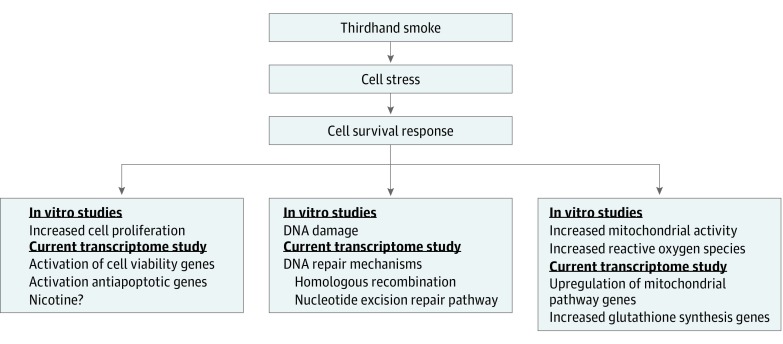
Schematic Diagram Summarizing the Responses of Human Nasal Epithelium to Thirdhand Smoke Thirdhand smoke induced cellular stress leading to activation of cell survival responses, including activation of DNA repair pathways, increased cell proliferation, and increased mitochondrial activity in the human nasal epithelium. Previous in vitro studies have shown similar results in which thirdhand smoke causes DNA damage,^9,10^ increased cell proliferation,^11,43^ increased mitochondria activity,^12^ and increased reactive oxygen species.^12^

In summary, this is the first exposure study to document an association between THS and gene expression in humans. Our results show that THS induced cell survival responses, which included upregulation of genes involved in DNA repair, activation of cell viability, increased mitochondrial activity, and inhibition of cell death (Figure 3). These changes are very similar to those reported previously for in vitro cultured cells.^9,11,12^ Importantly, the changes in gene expression in the current study were seen following a relatively short (3-hour) exposure, indicating that humans respond rapidly to THS. Future studies on long-term exposure in conjunction with our study could broaden our understanding of the effects of THS on human health. Our study provides an important foundation for physicians treating patients exposed to THS and for future development of regulations dealing with remediation of indoor environments contaminated with THS.

## Notes

Supplement 1. **Trial Protocol**

Supplement 2. **eFigure 1.** Experimental Design for Clean Air and THS Exposure **eTable 1.** Number of Differentially Expressed Genes (DEGs) for Clean Air and THS Exposure **eFigure 2.** Volcano Plots Showing the DEGs After Clean Air or THS Exposure **eTable 2.** Log_2_-Fold Change, *P*-Values, and FDR Values of DEGs After Clean Air Exposure **eTable 3.** Log_2_-Fold Change, *P*-Values, and FDR Values of DEGs After THS Exposure **eTable 4.** GO Terms: Biological Processes Enriched After THS Exposure **eTable 5.** GO Terms: Cellular Components Enriched After THS Exposure **eTable 6.** GO Terms: Molecular Function Enriched After THS Exposure **eTable 7.** Reactome Terms Enriched After THS Exposure **eFigure 3.** Diagram Showing the Cell Death Associated Genes That Were Upregulated Following THS Exposure in the IPA Analysis **eFigure 4.** Diagram Showing the Cell Viability Genes That Were Upregulated Following THS Exposure in the IPA Analysis **eFigure 5.** Diagram Showing the Homologous Recombination Genes That Were Upregulated Following THS Exposure in the IPA Analysis **eReferences**

Supplement 3. **Data Sharing Statement**
