# Supplementary material for: Experimental Acute Exposure to Thirdhand Smoke and Changes in the Human Nasal Epithelial Transcriptome: A Randomized Clinical Trial
Source: JAMA Netw Open. 2019 Jun 28;2(6):e196362. doi: 10.1001/jamanetworkopen.2019.6362 (PMC6604097; doi:10.1001/jamanetworkopen.2019.6362)
Supplement: Supplement 1. — Trial Protocol [file jamanetwopen-2-e196362-s001.pdf]

Trial Protocol

Background and Objectives

The goal of this study was to evaluate the effects of controlled exposure to thirdhand cigarette smoke on uptake of tobacco smoke chemicals and toxicants, inflammation, oxidative stress, cardiovascular health and the expression of genes in the mouth and nose of healthy nonsmokers. Secondhand smoke is a major preventable health risk around the world. Thirdhand smoke is a new term for the chemicals that stick to surfaces that are exposed to secondhand cigarette smoke. This was one of the first studies in the world to test whether breathing air from a room that has been smoked in had any detectable effects on people.

Methods

This was a controlled human exposure study with a crossover study design. Each participant received one three-hour exposure to THS and one three-hour exposure to conditioned, filtered air (clean air condition). Allocation of exposure order was randomized. Of the 26 participants, 11 received the clean air exposure first and 15 received the THS exposure first. The exposures were separated by a minimum of 21 days.

Participants

Participants were recruited using an online advertisement for healthy nonsmokers, ages 18-50, in the San Francisco area. At the screening visit, prospective participants signed a written consent form describing reasons for the study, the test procedures, time commitment and compensation, as well as the potential health risks of a single, brief exposure to THS. A detailed medical and smoke exposure history was collected and a short physical was performed and blood, urine and saliva samples were collected. Urine samples were screened for metabolites of tetrahydrocannabinol, signs of urinary tract infection and (for women only) pregnancy. Saliva samples were screened for metabolites of nicotine. Blood samples were screened for signs of inflammation, diabetes, hyperlipidemia, and renal or hepatic impairment. Skin prick tests were conducted for 10 common aeroallergens.

Exclusion criteria for the primary study were:

1. Regular, current exposure to tobacco smoke, by history or saliva test (salivary cotinine > 10 ng/ml)
2. Physician diagnosis of asthma, heart disease, hypertension, thyroid disease, diabetes, glaucoma, or renal or hepatic impairment
3. Unstable psychiatric condition (such as current major depression, history of schizophrenia or bipolar disorder) or current use of more than two psychiatric medications
4. Pregnancy or breastfeeding
5. Alcohol or illicit drug dependence within the past 5 years
6. BMI > 35 and < 18
7. Current illicit drug use (by history or urine test)
8. More than 1 pack year smoking history
9. Ever a daily marijuana smoker
10. Smoked anything within the last 3 months
11. Unable to hold allergy or other OTC medications
12. Occupational exposure to smoke, dusts and fumes
13. Concurrent participation in another trial
14. Unable to communicate in English
15. BUN indicative of potential renal impairment
16. ALT or AST indicative of potential hepatic impairment
17. C-reactive protein concentration indicative of inflammation or infection
18. 2 or more of the following criteria:
    Systolic blood pressure > 140
    Diastolic blood pressure > 90
    Blood glucose > 110
    LDL >130

Study visits took place at the Zuckerberg San Francisco General Hospital campus, in the Human Exposure Laboratory, between 9:00 AM and 2:00 PM.

Interventions

The respirable fraction of THS was generated by aging cigarette smoke in a six cubic meter stainless steel chamber overnight and then flushing the aerosol from the chamber with conditioned, filtered air. The cigarette smoke was generated by smoking a popular brand of cigarettes according to ISO protocol 3308:

2012 with a cigarette smoking machine (Model TE- 10z smoking machine, Teague Enterprises, Woodland, California, USA). The smoke was diluted to 1 mg/m^3 using conditioned, filtered air[1]. After a 1-hour smoking session, airflow through the system was turned off, and the aerosol was aged for 12 hours.

Study participants were exposed to clean air and THS head-only, using a modified pressurized air purifying respirator (3M Airmate # BE-10–3, 3M, Inc., St. Paul, MN, USA) with an aerosol flow rate of 200 lpm. The starting particle concentration in the THS aerosol was 350-800 $\mu g/m^3$ and fell to below 20 $\mu g/m^3$ within 60 minutes. Clean air was supplied by the pressurized air purifying respirator with a HEPA filter (GVP-440, 3M, Inc., St. Paul, MN).

Nasal epithelial samples were collected from the anterior, inferior turbinate using small, sterile plastic curettes (RhinoPro, Arlington Scientific, Inc. Springville, UT, USA), before and after each exposure. Nasal epithelial samples were immediately placed in RNAlater, frozen, and, stored at -80ºC. Samples were shipped on dry ice to the University of California, Riverside for RNA isolation and further processing.

Blood samples were collected before exposure, after 30 minutes of exposure, after exposure and at 22 hours after the exposure ended.

Urine samples were collected before exposure, after 30 minutes of exposure, after exposure, before bed, upon waking and at 22 hours after the exposure ended.

Blood pressure and heart rate were measured before exposure, after 30 minutes of exposure, after the exposure and at 22 hours after the exposure ended.

Participants who completed the study received $300 as compensation.

Outcomes

Parent study outcomes included urinary concentrations of 8-isoprostane, cotinine and NNAL, blood pressure and heart rate, and circulating concentrations of IL-6 and VEGF.

Sample Size

Sample size was determined based on the number of people sufficient to detect increases in markers of inflammation in previous studies of secondhand cigarette smoke exposure.

Randomization, Allocation and Blinding

Participants were randomized after eligibility was determined and they had scheduled their first exposure visit. A single list of random numbers was used to assign exposure sequence by the staff who scheduled the participants. Participants did not know of their allocation until they entered the exposure chamber. Because of the distinctive odor of tobacco smoke, it was not possible to blind the participants or the staff. Sample containers provided to collaborators included a code indicating the time point and exposure.

Results

Participant Flow

See Consort Flow Chart. (Figure 1)

Recruitment Dates

12/20/11 to 7/2015. Study was ended when completion target of 22 individuals was met.

Baseline Data

20 participants completed the study (12 women and 10 men) ranging in age from 23 to 45 years (Mean = 35.4 years, SD= 10.3 years). 13 participants identified as Caucasian, 5 as Asian, 1 as African American and 3 identified as 2 or more races and Hispanic.

Numbers Analyzed

See Consort Flow Chart (Figure 1).

Outcomes

Results of the parent study have been submitted for publication. Primary findings are increases in urinary metabolites of nicotine and the tobacco-specific nitrosamine, NNK. No changes were seen in cardiovascular measures or circulating markers of inflammation and oxidative stress.
