# Supplementary material for: Experimental Acute Exposure to Thirdhand Smoke and Changes in the Human Nasal Epithelial Transcriptome: A Randomized Clinical Trial
Source: JAMA Netw Open. 2019 Jun 28;2(6):e196362. doi: 10.1001/jamanetworkopen.2019.6362 (PMC6604097; doi:10.1001/jamanetworkopen.2019.6362)
Supplement: Supplement 2. — eFigure 1. Experimental Design for Clean Air and THS Exposure eTable 1. Number of Differentially Expressed Genes (DEGs) for Clean Air and THS Exposure eFigure 2. Volcano Plots Showing the DEGs After Clean Air or THS Exposure eTable 2. Log2-Fold Change, P-Values, and FDR Values of DEGs After Clean Air Exposure eTable 3. Log2-Fold Change, P-Values, and FDR Values of DEGs After THS Exposure eTable 4. GO Terms: Biological Processes Enriched After THS Exposure eTable 5. GO Terms: Cellular Components Enriched After THS Exposure eTable 6. GO Terms: Molecular Function Enriched After THS Exposure eTable 7. Reactome Terms Enriched After THS Exposure eFigure 3. Diagram Showing the Cell Death Associated Genes That Were Upregulated Following THS Exposure in the IPA Analysis eFigure 4. Diagram Showing the Cell Viability Genes That Were Upregulated Following THS Exposure in the IPA Analysis eFigure 5. Diagram Showing the Homologous Recombination Genes That Were Upregulated Following THS Exposure in the IPA Analysis eReferences [file jamanetwopen-2-e196362-s002.pdf]

Supplementary Online Content

Pozuelos GL, Kagda MS, Schick S, Girke T, Volz DC, Talbot P. Experimental acute exposure to thirdhand smoke and changes in the human nasal epithelial transcriptome: a randomized clinical trial. *JAMA Netw Open*. 2019;2(6):e196362. doi:10.1001/jamanetworkopen.2019.6362

eFigure 1. Experimental Design for Clean Air and THS Exposure
eTable 1. Number of Differentially Expressed Genes (DEGs) for Clean Air and THS Exposure
eFigure 2. Volcano Plots Showing the DEGs After Clean Air or THS Exposure
eTable 2. Log_2-Fold Change, *P*-Values, and FDR Values, and of DEGs After Clean Air Exposure
eTable 3. Log_2-Fold Change, *P*-Values, and FDR Values and of DEGs After THS Exposure
eTable 4. GO Terms: Biological Processes Enriched After THS Exposure
eTable 5. GO Terms: Cellular Components Enriched After THS Exposure
eTable 6. GO Terms: Molecular Function Enriched After THS Exposure
eTable 7. Reactome Terms Enriched After THS Exposure
eFigure 3. Diagram Showing the Cell Death Associated Genes That Were Upregulated Following THS Exposure in the IPA Analysis
eFigure 4. Diagram Showing the Cell Viability Genes That Were Upregulated Following THS Exposure in the IPA Analysis
eFigure 5. Diagram Showing the Homologous Recombination Genes That Were Upregulated Following THS Exposure in the IPA Analysis
eReferences

This supplementary material has been provided by the authors to give readers additional information about their work.

© 2019 Pozuelos GL et al. *JAMA Network Open.*

eFigure 1. Experimental Design for Clean Air and THS Exposure. Schematic diagram illustrating experimental design in which nasal scraps were taken before and after exposure to either clean air (A) or THS (B) after a 3 hour exposure.

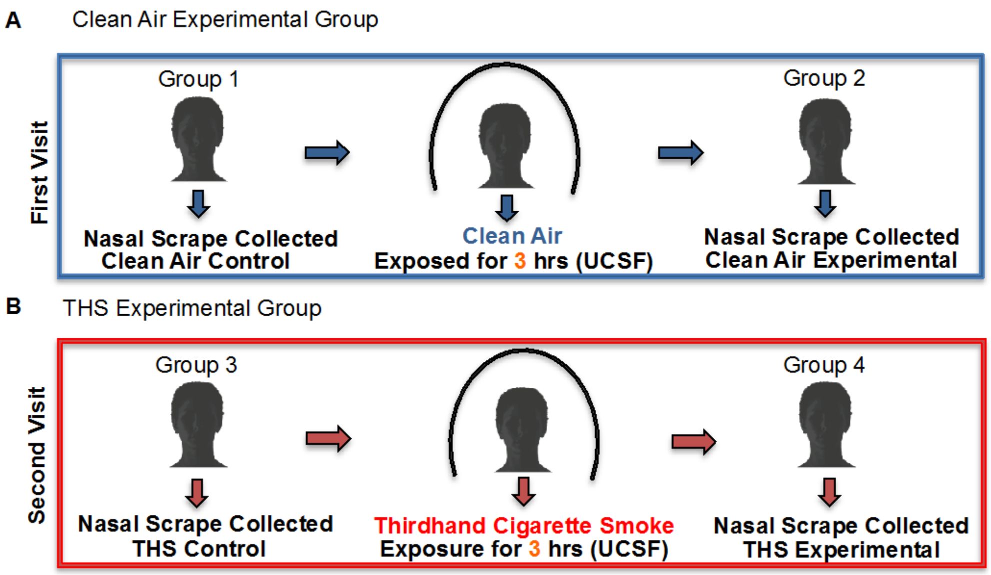

eTable 1. Number of Differentially Expressed Genes (DEGs) for Clean Air and THS Exposure.

	Down Regulated DEGs	Up-Regulated DEGs
**Clean Air Exposure**	0	2
**THS Exposure**	7	382

© 2019 Pozuelos GL et al. *JAMA Network Open.*

eFigure 2. Volcano Plots Showing the DEGs After Clean Air or THS Exposure.

(A) DEGs following clean air exposure and (B) following THS exposure. Blue dots represent genes that were not significant (FDR > 0.1), and magenta dots represent DEGs (FDR < 0.1).

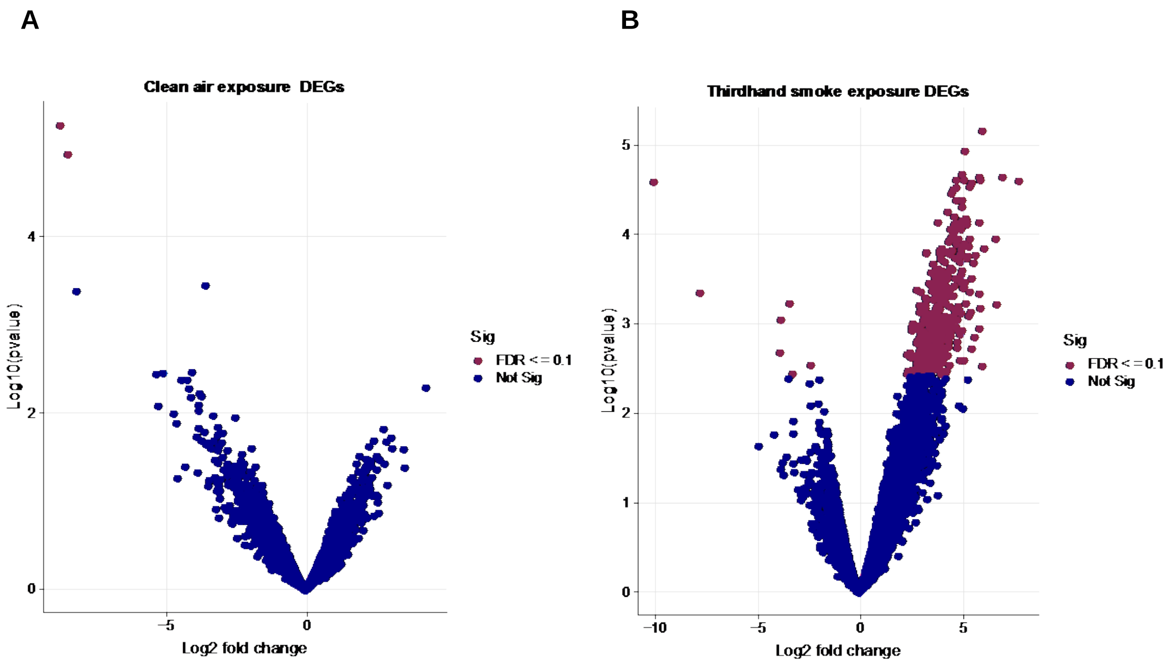

eTable 2. Log2-Fold Change, P-Values, and FDR Values, and of DEGs After Clean Air Exposure.

Genes	Log2 -Fold Change	P-Value	FDR
HBA2	-8.71	5.38E-06	0.06
HBA1	-8.41	1.13E-05	0.06

© 2019 Pozuelos GL et al. *JAMA Network Open.*

eTable 3. Log_2 -Fold Change, P-Values, and FDR values and of DEGs After THS Exposure.

Genes	Log_2 -Fold Change	P-Value	FDR
ANAPC11	5.00	2.06E-05	0.02
CIB1	5.16	1.14E-05	0.02
COMT	4.61	3.07E-05	0.02
COPE	5.10	2.40E-05	0.02
ECH1	4.75	2.92E-05	0.02
HBG1	-9.96	2.54E-05	0.02
MAN1B1	6.02	6.83E-06	0.02
OSM	7.78	2.49E-05	0.02
PHPT1	5.47	2.61E-05	0.02
PPP5C	5.92	2.43E-05	0.02
TCEB2	4.76	2.39E-05	0.02
TFF3	7.01	2.25E-05	0.02
TMEM190	5.88	2.24E-05	0.02
ZMYND10	5.39	2.84E-05	0.02
ALDOA	4.52	3.43E-05	0.02
BRMS1	5.03	4.06E-05	0.03
PPP1CA	4.72	4.05E-05	0.03
AGTRAP	4.99	7.33E-05	0.03
ASL	5.14	7.25E-05	0.03
C19orf70	5.24	7.05E-05	0.03
NDUFV1	5.03	6.85E-05	0.03
PMPCA	5.87	7.29E-05	0.03
POLD2	5.21	6.53E-05	0.03
POLR2E	4.77	7.47E-05	0.03
POLR2I	4.65	7.55E-05	0.03
POLR2L	4.67	6.28E-05	0.03
R3HCC1	5.27	7.63E-05	0.03
ROMO1	4.31	5.46E-05	0.03
SNRPB	3.86	7.16E-05	0.03
TPPP3	5.04	4.82E-05	0.03
BSG	3.95	1.61E-04	0.03
BUD31	3.95	1.49E-04	0.03
C16orf71	4.96	1.43E-04	0.03
CCDC101	5.22	1.32E-04	0.03
CD151	4.03	1.43E-04	0.03
CENPM	6.64	1.09E-04	0.03
CFL1	3.28	1.59E-04	0.03
CHID1	4.76	1.25E-04	0.03
COMMD4	4.46	1.62E-04	0.03
DDX39A	4.95	1.67E-04	0.03
ETFB	4.45	8.69E-05	0.03
EXOSC5	6.12	1.43E-04	0.03
GPS1	4.99	1.63E-04	0.03
LGALS1	4.91	1.62E-04	0.03
MGMT	4.74	8.28E-05	0.03
MRPL28	4.99	8.66E-05	0.03
MVP	4.06	1.41E-04	0.03
NDUFA11	4.73	1.13E-04	0.03
NOC2L	5.35	1.34E-04	0.03

© 2019 Pozuelos GL et al. *JAMA Network Open.*

Genes	Log_2 -Fold Change	P-Value	FDR
OTUB1	4.28	1.52E-04	0.03
PMEL	5.68	1.69E-04	0.03
PMF1-BGLAP	5.13	1.25E-04	0.03
PRPF6	4.39	1.17E-04	0.03
PTCD1	4.37	1.59E-04	0.03
RPL18	4.56	1.03E-04	0.03
SCRN2	5.29	1.70E-04	0.03
STRA13	4.53	1.50E-04	0.03
TARBP2	4.82	1.10E-04	0.03
TCF25	4.44	1.11E-04	0.03
TMEM265	3.29	1.56E-04	0.03
TOM1	4.31	1.55E-04	0.03
TRAPPC6A	5.00	1.33E-04	0.03
TSTA3	5.39	1.11E-04	0.03
VPS28	4.64	9.25E-05	0.03
WBP2	4.09	1.55E-04	0.03
ADGRE5	3.99	1.83E-04	0.03
ARL2	5.27	1.80E-04	0.03
C12orf57	4.71	1.83E-04	0.03
MAGED2	3.99	1.83E-04	0.03
SCAMP2	4.17	2.00E-04	0.03
SSSCA1	4.93	2.00E-04	0.03
CLK3	5.60	2.04E-04	0.03
WWP2	3.63	2.11E-04	0.03
AP2S1	4.43	2.30E-04	0.03
IDH3G	4.55	2.40E-04	0.03
KAT5	4.42	2.41E-04	0.03
LYPD2	3.84	2.39E-04	0.03
VMO1	3.76	2.41E-04	0.03
NHP2	4.06	2.50E-04	0.03
NRBP1	3.95	2.48E-04	0.03
TBRG4	5.00	2.53E-04	0.03
OAZ1	3.51	2.65E-04	0.03
PCIF1	4.34	2.68E-04	0.03
TEN1	4.98	2.73E-04	0.03
MTX1	4.05	2.77E-04	0.04
AAMP	3.81	2.99E-04	0.04
ARPC4	3.86	2.90E-04	0.04
CD82	3.71	2.99E-04	0.04
CDC42EP2	4.90	3.10E-04	0.04
MRPS11	4.26	3.11E-04	0.04
NDUFB7	4.34	2.96E-04	0.04
NDUFS8	4.14	2.94E-04	0.04
TEN1-CDK3	4.92	3.08E-04	0.04
YIPF2	4.71	3.07E-04	0.04
ZBTB48	5.47	3.08E-04	0.04
C19orf33	3.53	3.58E-04	0.04
CCDC103	4.13	3.50E-04	0.04
CRIP1	3.98	3.52E-04	0.04
KRT8	3.69	3.55E-04	0.04
LGALS3BP	3.77	3.48E-04	0.04
LINC00116	5.14	3.39E-04	0.04

© 2019 Pozuelos GL et al. *JAMA Network Open.*

Genes	Log_2 -Fold Change	P-Value	FDR
MT2A	4.10	3.29E-04	0.04
PPAP2C	3.56	3.43E-04	0.04
WDR25	4.98	3.34E-04	0.04
YIF1B	3.44	3.48E-04	0.04
NAPA	4.00	3.66E-04	0.04
ALOX15P1	5.13	3.81E-04	0.04
GMPPA	4.67	3.75E-04	0.04
LMAN2	4.00	3.84E-04	0.04
SGSM3	4.26	3.77E-04	0.04
BCKDHA	4.45	3.96E-04	0.04
PRMT7	5.05	3.93E-04	0.04
PRSS8	4.26	3.99E-04	0.04
RPL28	3.88	4.02E-04	0.04
UBAC2	3.90	3.99E-04	0.04
FTSJ1	2.81	4.06E-04	0.04
ACO2	3.73	4.32E-04	0.04
C12orf10	4.38	4.22E-04	0.04
DPM3	5.39	4.27E-04	0.04
LYPLA2	4.13	4.31E-04	0.04
NDUFC2-KCTD14	3.00	4.32E-04	0.04
UBL7	4.25	4.15E-04	0.04
ZNHIT1	3.58	4.24E-04	0.04
ENTPD6	4.35	4.43E-04	0.04
HBG2	-7.74	4.44E-04	0.04
PMF1	4.07	4.38E-04	0.04
MIR4761	5.87	4.50E-04	0.04
ASCC2	3.93	4.85E-04	0.04
CHCHD5	4.59	4.72E-04	0.04
KIAA0195	4.09	4.83E-04	0.04
RUVBL1	3.75	4.75E-04	0.04
ZMAT5	4.47	4.78E-04	0.04
TIMM13	4.05	4.93E-04	0.04
PIGT	3.24	5.03E-04	0.04
ISG20	4.10	5.26E-04	0.04
TMEM205	3.85	5.14E-04	0.04
TNFSF12-TNFSF13	3.64	5.23E-04	0.04
TNFSF13	3.30	5.20E-04	0.04
RPP21	4.19	5.30E-04	0.04
ADGRG6	-3.36	5.74E-04	0.04
PEX16	5.20	5.81E-04	0.04
SCAMP3	4.24	5.81E-04	0.04
ST3GAL4-AS1	6.70	5.89E-04	0.04
GALE	3.82	6.18E-04	0.04
HIGD2A	3.18	6.12E-04	0.04
HN1	3.26	6.12E-04	0.04
NDUFB2	2.94	6.15E-04	0.04
SLC35C2	3.91	6.06E-04	0.04
WRAP53	4.40	6.18E-04	0.04
CATSPERD	4.84	6.27E-04	0.04
USP11	3.79	6.40E-04	0.05
ABHD11-AS1	5.90	6.63E-04	0.05
ARPC4-TTLL3	3.85	6.68E-04	0.05

© 2019 Pozuelos GL et al. *JAMA Network Open.*

Genes	Log_2 -Fold Change	P-Value	FDR
CLN3	3.75	6.76E-04	0.05
CNN2	3.74	6.51E-04	0.05
DCPS	3.87	6.74E-04	0.05
SIL1	3.93	6.66E-04	0.05
TAX1BP3	3.25	6.64E-04	0.05
TSSC4	4.63	6.74E-04	0.05
DDX49	4.90	6.87E-04	0.05
HIST1H2BC	4.69	7.19E-04	0.05
H3F3C	2.94	7.30E-04	0.05
SDSL	5.46	7.32E-04	0.05
RPL18A	3.58	7.40E-04	0.05
CHCHD6	4.96	7.48E-04	0.05
GSS	3.18	7.60E-04	0.05
LRG1	3.31	7.60E-04	0.05
TEX264	3.85	7.86E-04	0.05
MEF2BNB-MEF2B	5.35	7.95E-04	0.05
TIMM17B	4.17	8.12E-04	0.05
VPS39	3.74	8.09E-04	0.05
PAFAH1B3	4.43	8.26E-04	0.05
PARP3	3.42	8.24E-04	0.05
PCNA-AS1	4.64	8.34E-04	0.05
SNAP47	3.64	8.33E-04	0.05
ANAPC5	3.21	8.39E-04	0.05
NANS	3.22	8.57E-04	0.05
TADA3	3.39	8.65E-04	0.05
USB1	3.52	8.62E-04	0.05
CYB561A3	3.37	8.79E-04	0.05
RPL36	3.14	8.79E-04	0.05
TEKT2	4.78	8.85E-04	0.05
LINC01224	-3.78	8.93E-04	0.05
RPP25L	4.31	9.00E-04	0.05
ANKZF1	3.96	9.13E-04	0.05
CHI3L1	3.65	9.19E-04	0.05
GPR162	3.77	9.21E-04	0.05
GSTP1	3.66	9.06E-04	0.05
AEN	4.22	9.46E-04	0.05
CXXC1	4.53	9.47E-04	0.05
DNAL4	3.91	9.40E-04	0.05
LSP1	4.10	9.47E-04	0.05
SIRT2	3.73	9.45E-04	0.05
ARF1	3.17	9.54E-04	0.05
EWSR1	2.62	9.69E-04	0.05
NARF	3.97	9.72E-04	0.05
SF3B4	3.50	9.68E-04	0.05
ST3GAL4	3.42	9.87E-04	0.05
RAD9A	3.93	1.00E-03	0.05
EIF4H	2.89	1.03E-03	0.06
ALG12	4.46	1.06E-03	0.06
NDUFC2	2.56	1.06E-03	0.06
RSPH9	3.86	1.06E-03	0.06
TUBG1	3.40	1.04E-03	0.06
CCDC57	4.73	1.08E-03	0.06

© 2019 Pozuelos GL et al. *JAMA Network Open.*

Genes	Log_2 -Fold Change	P-Value	FDR
DUOXA1	3.49	1.09E-03	0.06
LOC105447645	4.26	1.09E-03	0.06
TECR	3.87	1.09E-03	0.06
TMED1	4.38	1.09E-03	0.06
ACOT8	4.33	1.12E-03	0.06
CDK10	5.85	1.11E-03	0.06
NDUFB2-AS1	4.20	1.12E-03	0.06
LRRC46	3.58	1.13E-03	0.06
AP4M1	4.02	1.15E-03	0.06
HIST2H4A	5.03	1.15E-03	0.06
HIST2H4B	5.03	1.15E-03	0.06
SEC61B	2.58	1.16E-03	0.06
SNCG	4.82	1.17E-03	0.06
U2AF1	2.86	1.14E-03	0.06
UQCR11	3.04	1.16E-03	0.06
STK36	3.58	1.18E-03	0.06
PPP1R32	4.89	1.19E-03	0.06
MUC20	3.15	1.19E-03	0.06
FAM195B	4.45	1.21E-03	0.06
ANPEP	3.26	1.26E-03	0.06
DMAP1	4.46	1.25E-03	0.06
IFI30	3.12	1.27E-03	0.06
NCF4	3.85	1.25E-03	0.06
NOL12	4.35	1.27E-03	0.06
C11orf49	3.23	1.28E-03	0.06
CCDC159	4.91	1.28E-03	0.06
C6orf1	4.03	1.29E-03	0.06
XRCC1	3.90	1.30E-03	0.06
DNPEP	2.99	1.31E-03	0.06
RAB5C	3.13	1.31E-03	0.06
ABHD16A	3.19	1.34E-03	0.06
C19orf48	4.46	1.34E-03	0.06
ELOF1	3.94	1.41E-03	0.06
GPX3	3.52	1.41E-03	0.06
HEXA	3.41	1.39E-03	0.06
LYPLA2P2	5.64	1.38E-03	0.06
PPARD	3.65	1.38E-03	0.06
RAD23A	3.23	1.41E-03	0.06
SLC16A5	3.27	1.39E-03	0.06
TAPBPL	3.51	1.40E-03	0.06
TBCD	3.95	1.38E-03	0.06
AK1	3.87	1.44E-03	0.06
CINP	4.26	1.44E-03	0.06
PPP2R1A	3.24	1.45E-03	0.06
RPS5	3.21	1.45E-03	0.06
XPC	3.25	1.43E-03	0.06
GCHFR	3.87	1.46E-03	0.06
MX1	2.93	1.46E-03	0.06
LAPTM5	3.08	1.47E-03	0.06
BIN3	3.65	1.50E-03	0.06
ARAF	3.36	1.53E-03	0.06
FLOT2	3.27	1.53E-03	0.06

© 2019 Pozuelos GL et al. *JAMA Network Open.*

Genes	Log_2 -Fold Change	P-Value	FDR
BSCL2	3.39	1.54E-03	0.06
GAS8	3.85	1.55E-03	0.06
GPRC5C	3.26	1.57E-03	0.06
HS1BP3	4.78	1.58E-03	0.06
MLST8	4.01	1.56E-03	0.06
P2RX5-TAX1BP3	2.84	1.57E-03	0.06
ZNF576	4.35	1.56E-03	0.06
COX8A	2.80	1.60E-03	0.06
GRIPAP1	3.00	1.61E-03	0.06
NADSYN1	3.81	1.64E-03	0.07
NCAPH2	3.79	1.65E-03	0.07
SZRD1	3.16	1.65E-03	0.07
CYP2F1	3.07	1.69E-03	0.07
DDX41	3.77	1.69E-03	0.07
PHGDH	3.74	1.68E-03	0.07
SPTAN1	3.11	1.72E-03	0.07
STK11IP	4.38	1.77E-03	0.07
CARKD	3.27	1.78E-03	0.07
MUL1	3.57	1.79E-03	0.07
CCS	4.77	1.80E-03	0.07
KIF22	4.15	1.82E-03	0.07
MED24	3.37	1.82E-03	0.07
CDC37	3.63	1.83E-03	0.07
WFDC2	3.18	1.83E-03	0.07
BCL2L1	3.18	1.87E-03	0.07
C9orf114	3.54	1.87E-03	0.07
MFAP2	5.50	1.85E-03	0.07
RPL29	3.05	1.86E-03	0.07
ZDHHC12	4.11	1.86E-03	0.07
CPSF3L	3.84	1.88E-03	0.07
RPS9	3.14	1.88E-03	0.07
WDR55	3.11	1.90E-03	0.07
RABGGTA	3.54	1.92E-03	0.07
BLVRB	3.38	1.93E-03	0.07
DAGLB	4.13	1.94E-03	0.07
PHB	3.21	1.94E-03	0.07
HNRNPUL2-BSCL2	3.28	1.95E-03	0.07
ARHGAP1	2.97	1.97E-03	0.07
EXOC7	2.98	1.98E-03	0.07
TREX1	3.80	1.98E-03	0.07
MRPL53	4.09	2.00E-03	0.07
PRKCSH	3.60	2.00E-03	0.07
LOC101928223	-3.83	2.04E-03	0.07
GSTO1	2.48	2.04E-03	0.07
NUP188	4.19	2.08E-03	0.07
MRPL52	3.17	2.11E-03	0.07
TIMM10B	2.50	2.13E-03	0.08
MRPL55	3.56	2.15E-03	0.08
NTMT1	3.81	2.16E-03	0.08
PLEKHJ1	3.76	2.16E-03	0.08
SGTA	3.99	2.17E-03	0.08
CCDC37	3.64	2.18E-03	0.08

© 2019 Pozuelos GL et al. *JAMA Network Open.*

Genes	Log_2 -Fold Change	P-Value	FDR
RBM23	2.34	2.19E-03	0.08
WDR45	3.31	2.21E-03	0.08
GPATCH3	4.21	2.24E-03	0.08
SRA1	2.76	2.24E-03	0.08
TFPT	4.32	2.24E-03	0.08
ITFG3	4.05	2.26E-03	0.08
LRPAP1	3.30	2.27E-03	0.08
NAP1L4	2.74	2.27E-03	0.08
COASY	3.33	2.32E-03	0.08
HLA-E	2.55	2.31E-03	0.08
URM1	3.36	2.32E-03	0.08
ARL16	3.13	2.36E-03	0.08
RNASEH2C	3.24	2.38E-03	0.08
DPP3	2.95	2.41E-03	0.08
ST20	3.93	2.41E-03	0.08
MSRB1	3.28	2.44E-03	0.08
ACY1	3.77	2.49E-03	0.08
LENG1	4.94	2.49E-03	0.08
RRP12	4.11	2.50E-03	0.08
ABHD14A-ACY1	3.69	2.54E-03	0.08
CLCNKB	5.10	2.53E-03	0.08
CXCL8	3.41	2.52E-03	0.08
EEF1D	3.07	2.54E-03	0.08
KRT17	3.25	2.52E-03	0.08
COG7	3.26	2.63E-03	0.08
DCP1B	3.56	2.63E-03	0.08
HMBS	3.45	2.61E-03	0.08
LOC102724814	3.90	2.61E-03	0.08
PDZK1IP1	3.71	2.62E-03	0.08
SARS2	4.06	2.62E-03	0.08
VSIG2	3.01	2.59E-03	0.08
WDR86-AS1	3.35	2.63E-03	0.08
POMT1	4.06	2.64E-03	0.08
LAMTOR4	3.35	2.66E-03	0.08
ZER1	3.17	2.68E-03	0.08
ABT1	3.06	2.70E-03	0.08
GLIPR2	2.82	2.71E-03	0.08
PSMG3	2.84	2.71E-03	0.08
PRMT1	3.43	2.75E-03	0.08
LIN37	4.43	2.85E-03	0.09
MMP3	-2.32	2.85E-03	0.09
SSNA1	3.45	2.85E-03	0.09
TXLNA	3.09	2.85E-03	0.09
DRG2	3.80	2.91E-03	0.09
EMP3	5.98	2.95E-03	0.09
NFYC	3.03	3.03E-03	0.09
SLC25A11	2.88	3.05E-03	0.09
POLR3H	2.87	3.08E-03	0.09
SLC44A4	3.11	3.10E-03	0.09
EIF3C	2.77	3.11E-03	0.09
EIF3CL	2.77	3.12E-03	0.09
THAP3	3.70	3.16E-03	0.09

© 2019 Pozuelos GL et al. *JAMA Network Open.*

Genes	Log_2-Fold Change	P-Value	FDR
BCL2A1	2.92	3.19E-03	0.09
CABP7	3.92	3.21E-03	0.09
DIABLO	2.62	3.21E-03	0.09
PISD	2.66	3.23E-03	0.09
SLC35A4	3.28	3.23E-03	0.09
CRELD1	4.08	3.26E-03	0.10
ABTB1	4.25	3.28E-03	0.10
ARFGAP2	3.35	3.31E-03	0.10
EIF3G	3.50	3.31E-03	0.10
SERPINF1	3.07	3.30E-03	0.10
UQCRQ	2.41	3.30E-03	0.10
COA3	2.56	3.39E-03	0.10
FBXO6	3.44	3.39E-03	0.10
NEU1	2.45	3.39E-03	0.10
PSMB10	2.86	3.36E-03	0.10
TPD52L2	3.33	3.39E-03	0.10
ZFYVE19	3.49	3.42E-03	0.10
TMEM219	2.70	3.46E-03	0.10
HCK	4.24	3.48E-03	0.10
C3orf30	-3.23	3.53E-03	0.10
IFI27L2	3.39	3.53E-03	0.10
TMEM9	3.02	3.54E-03	0.10
LGALS3	2.35	3.57E-03	0.10
NUDCD3	3.39	3.57E-03	0.10
PWP2	4.00	3.58E-03	0.10
SAE1	2.65	3.59E-03	0.10
HDAC11	4.19	3.62E-03	0.10
TMEM63A	3.52	3.61E-03	0.10

eTable 4. GO Terms: Biological Processes Enriched After THS Exposure

ID	Description	Gene Count	q-Value
GO:0045333	Cellular Respiration	15	2.39E-03
GO:0022904	Respiratory Electron Transport Chain	11	2.84E-03
GO:0042775	Mitochondrial ATP Synthesis Coupled Electron Transport	10	2.84E-03
GO:0042773	ATP Synthesis Coupled Electron Transport	10	2.84E-03
GO:0022613	Ribonucleoprotein Complex Biogenesis	24	4.19E-03
GO:0022900	Electron Transport Chain	13	6.97E-03
GO:0006605	Protein Targeting	21	6.97E-03
GO:0006120	Mitochondrial Electron Transport, NADH to Ubiquinone	7	6.97E-03
GO:0034470	ncRNA Processing	21	6.97E-03
GO:0006119	Oxidative Phosphorylation	10	7.24E-03
GO:0006364	rRNA Processing	16	7.24E-03

© 2019 Pozuelos GL et al. *JAMA Network Open.*

eTable 5. GO Terms: Cellular Components Enriched After THS Exposure

ID	Description	Gene Count	q-Value
GO:0005743	Mitochondrial Inner Membrane	29	7.21E-06
GO:0098798	Mitochondrial Protein Complex	14	8.30E-05
GO:0070469	Respiratory Chain	11	8.30E-05
GO:0098800	Inner Mitochondrial Membrane Protein Complex	12	2.36E-04
GO:0044455	Mitochondrial Membrane Part	15	2.77E-04
GO:0098803	Respiratory Chain Complex	8	4.38E-03
GO:1990204	Oxidoreductase Complex	9	4.76E-03
GO:0005746	Mitochondrial Respiratory Chain	8	5.60E-03
GO:0005747	Mitochondrial Respiratory Chain Complex I	6	5.60E-03
GO:0030964	NADH Dehydrogenase Complex	6	5.60E-03
GO:0045271	Respiratory Chain Complex I	6	5.60E-03
GO:0044391	Ribosomal Subunit	12	5.60E-03
GO:0015934	Large Ribosomal Subunit	9	8.75E-03

eTable 6. GO Terms: Molecular Function Enriched After THS exposure

ID	Description	Gene Count	q-Value
GO:0003954	NADH Dehydrogenase Activity	7	3.80E-03

© 2019 Pozuelos GL et al. *JAMA Network Open.*

eTable 7. Reactome Terms Enriched After THS exposure.

ID	Description	Gene Count	q-Value
R-HSA-1428517	The Citric Acid (TCA) Cycle and Respiratory Electron Transport	14	9.27E-03
R-HSA-611105	Respiratory Electron Transport	10	1.01E-02
R-HSA-72766	Translation	18	1.01E-02
R-HSA-1268020	Mitochondrial Protein Import	8	1.01E-02
R-HSA-72165	mRNA Splicing - Minor Pathway	7	1.05E-02
R-HSA-5696398	Nucleotide Excision Repair	10	1.05E-02
R-HSA-168273	Influenza Viral RNA Transcription and Replication	11	1.05E-02
R-HSA-73894	DNA Repair	18	1.05E-02
R-HSA-156827	L13a-mediated Translational Silencing of Ceruloplasmin Expression	10	1.05E-02
R-HSA-72706	GTP Hydrolysis and Joining of the 60S Ribosomal Subunit	10	1.05E-02
R-HSA-168255	Influenza Life Cycle	11	1.23E-02
R-HSA-72613	Eukaryotic Translation Initiation	10	1.23E-02
R-HSA-72737	Cap-dependent Translation Initiation	10	1.23E-02
R-HSA-446203	Asparagine N-linked Glycosylation	17	1.23E-02
R-HSA-8868773	rRNA Processing in the Nucleus and Cytosol	13	1.23E-02
R-HSA-163200	Respiratory Electron Transport, ATP Synthesis by Chemiosmotic Coupling, and Heat Production by Uncoupling Proteins.	10	1.23E-02
R-HSA-72689	Formation of a Pool of Free 40S Subunits	9	1.36E-02
R-HSA-72312	rRNA Processing	13	1.69E-02
R-HSA-168254	Influenza Infection	11	1.69E-02
R-HSA-6791226	Major Pathway of rRNA Processing in the Nucleolus and Cytosol	12	2.12E-02
R-HSA-156842	Eukaryotic Translation Elongation	8	2.80E-02
R-HSA-6799198	Complex I biogenesis	6	3.27E-02
R-HSA-5696394	DNA Damage Recognition in GG-NER	5	3.51E-02
R-HSA-203927	MicroRNA (miRNA) biogenesis	4	3.61E-02
R-HSA-446193	Biosynthesis of the N-glycan Precursor (dolichol lipid-linked oligosaccharide, LLO) and Transfer to a Nascent Protein	7	3.61E-02

© 2019 Pozuelos GL et al. *JAMA Network Open.*

eFigure 3. Diagram Showing the Cell Death Associated Genes that were Upregulated Following THS Exposure in the IPA Analysis.

Blue dotted lines indicate inhibition of cell death and yellow dotted lines indicate inconsistent findings to the predicted inhibition of Cell Death (z-score = -3.03). Grey lines indicate insufficient information to make a prediction. IPA predicted subcellular location (extracellular, plasma membrane, cytoplasm or nuclear) of each gene is shown.

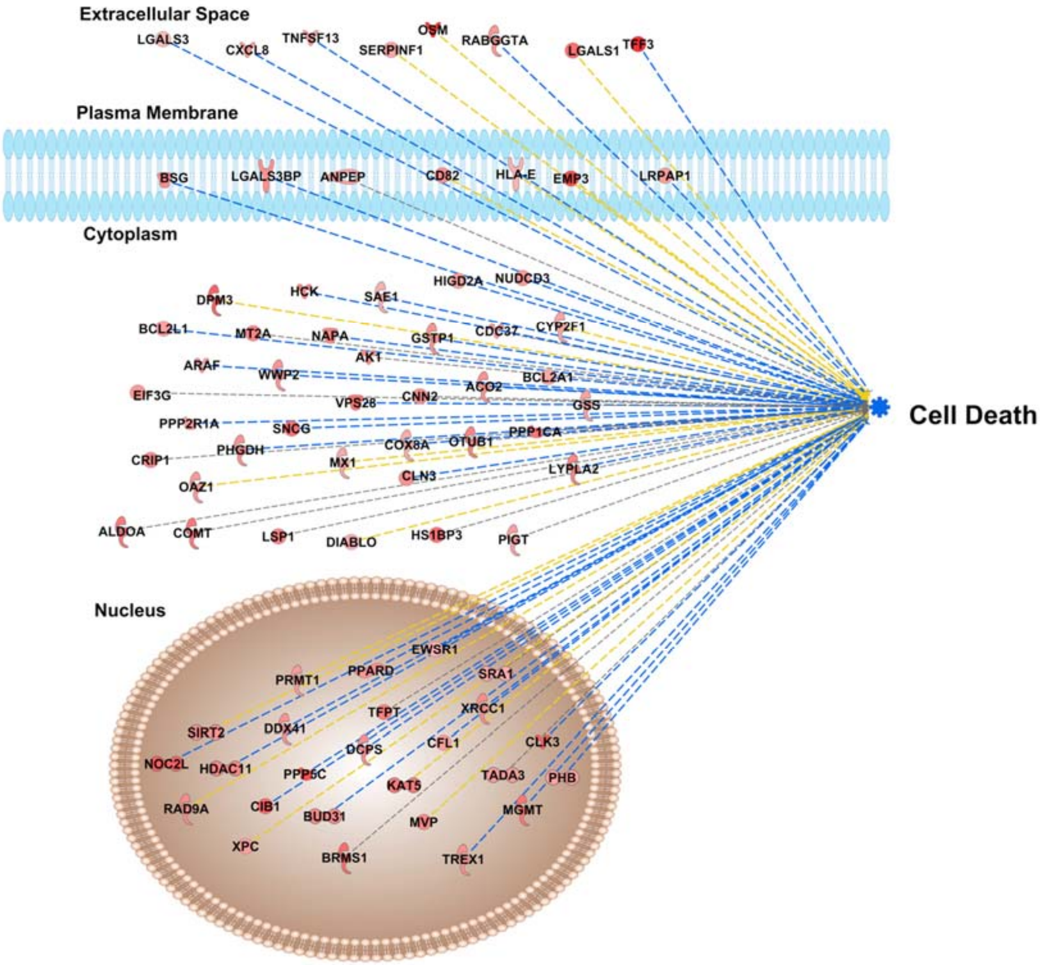

© 2019 Pozuelos GL et al. *JAMA Network Open.*

eFigure 4. Diagram Showing the Cell Viability Genes that were Upregulated Following THS Exposure in the IPA Analysis.

Orange dotted lines indicate activation of the process and yellow dotted lines indicate findings inconsistent with activation of cell viability (Z-score = 5.03). Predicted location of each gene (extracellular, plasma membrane, cytoplasm or nuclear) is shown.

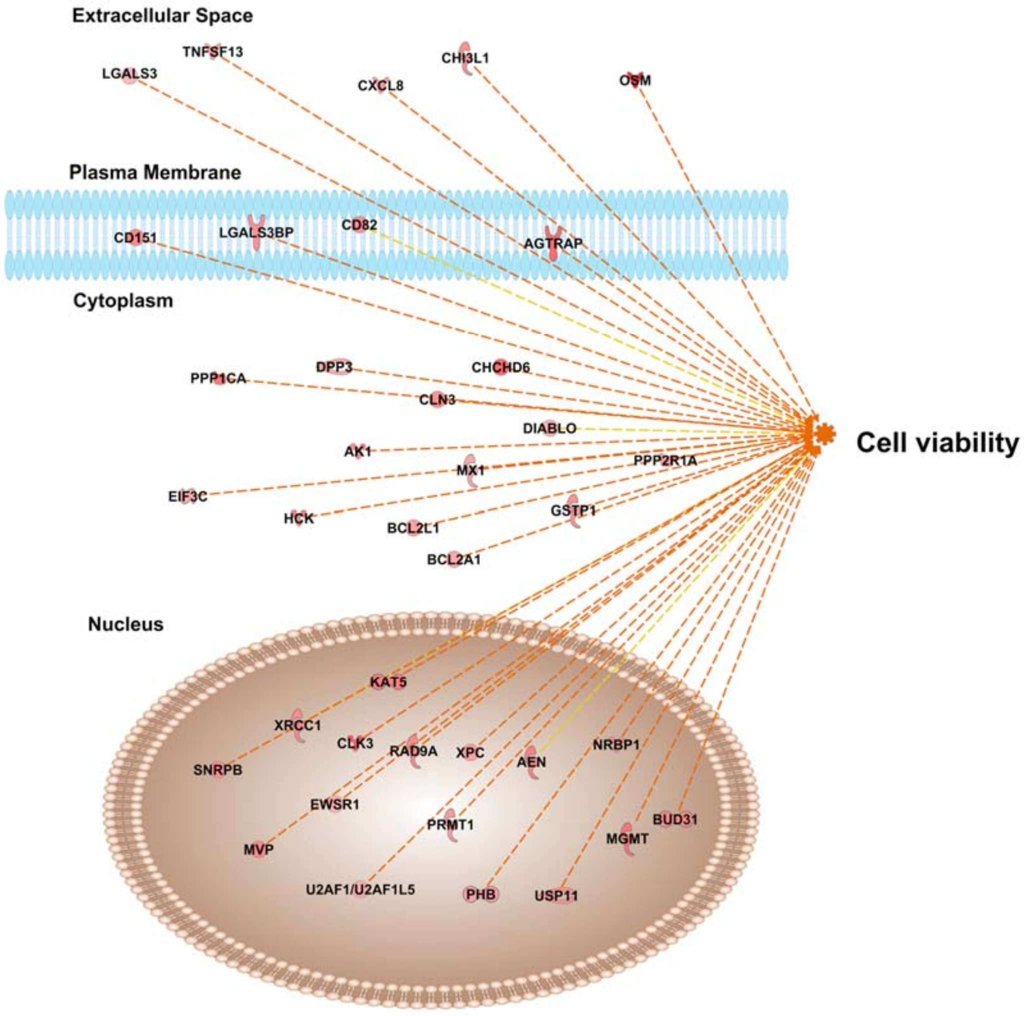

© 2019 Pozuelos GL et al. *JAMA Network Open.*

eFigure 5. Diagram Showing the Homologous Recombination Genes that were Upregulated Following THS Exposure in the IPA Analysis.

Orange dotted lines indicate activation of the process (z-score = 2.83). IPA based subcellular predicted location (extracellular, plasma membrane, cytoplasm or nuclear) of each gene is shown in the diagram.

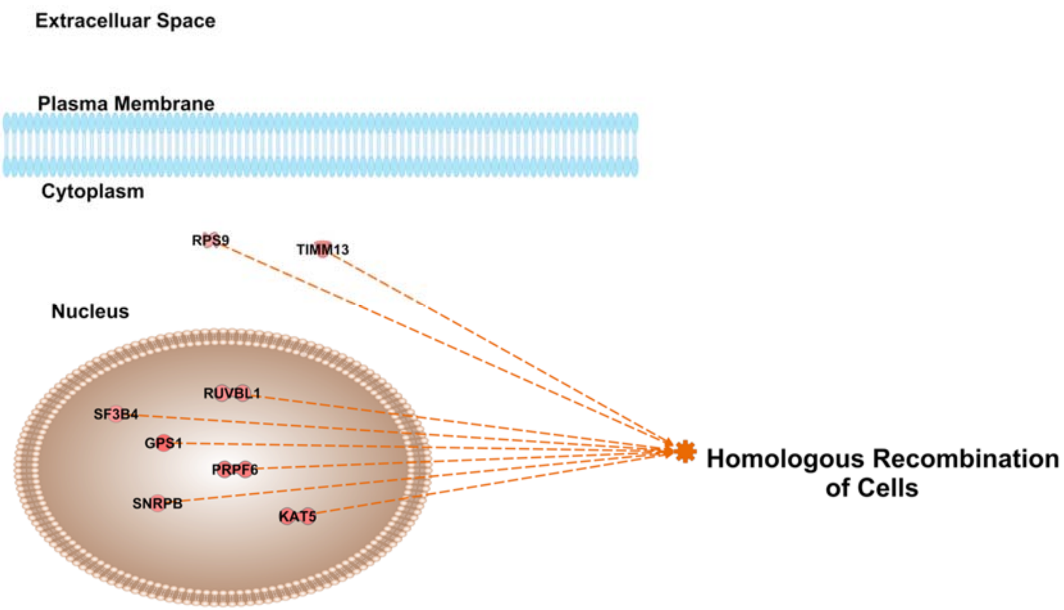

eReferences

1. Schick SF, Farraro KF, Fang J, et al. An apparatus for generating aged cigarette smoke for controlled human exposure studies. *Aerosol Science and Technology.* 2012;46(11):1246–1255.doi:10.1080/02786826.2012.708947.

© 2019 Pozuelos GL et al. *JAMA Network Open.*
