# Supplementary material for: Experimental Acute Exposure to Thirdhand Smoke and Changes in the Human Nasal Epithelial Transcriptome: A Randomized Clinical Trial
Source: JAMA Netw Open. 2019 Jun 28;2(6):e196362. doi: 10.1001/jamanetworkopen.2019.6362 (PMC6604097; doi:10.1001/jamanetworkopen.2019.6362)
Supplement: Supplement 3. — Data Sharing Statement [file jamanetwopen-2-e196362-s003.pdf]

Data Sharing Statement

Pozuelos GL, Kagda MS, Schick S, Girke T, Volz DC, Talbot P. Experimental acute exposure to thirdhand smoke and changes in the human nasal epithelial transcriptome: a randomized clinical trial. JAMA Netw Open. 2019;2(6):e196362. doi:10.1001/jamanetworkopen.2019.6362

Data
Data available: Yes
Data types: Other (please specify)
Additional Information: RNA-seq data (fastq files) have been uploaded to SRA and GEO.
How to access data: The data have been deposited in NCBI's BioProject database and are available through the SRA accession number PRJNA514351 (https://www.ncbi.nlm.nih.gov/sra/PRJNA514351) and GEO accession GSE129959
When available: With publication

Supporting Documents
Document types: None

Additional Information
Who can access the data: Anyone requesting the data.
Types of analyses: For any purpose.
Mechanisms of data availability: NCBI's bio-project database. Data are will be available without the investigators consent.
Any additional restrictions: none
